# Cerebrovascular–CSF coupling measured by broadband near-infrared spectroscopy as a physiological marker of brain aging and Alzheimer’s disease

**DOI:** 10.3389/fnagi.2026.1757765

**Published:** 2026-05-01

**Authors:** Fiza Saeed, Kathy L. Siepker, Soeun Jang, Sadra Shahdadian, Hanli Liu

**Affiliations:** 1 Department of Bioengineering, University of Texas at Arlington, Arlington, TX, United States; 2 School of Social Work, University of Texas at Arlington, Arlington, TX, United States

**Keywords:** Alzheimer’s disease, biomarker, broadband NIRS, cerebrospinal fluid, cerebrovascular-CSF coupling, near-infrared spectroscopy, total hemoglobin concentration

## Abstract

**Introduction:**

Alzheimer’s disease (AD) is strongly associated with cerebrovascular dysfunction and impaired glymphatic clearance. These dysfunctions may precede, contribute to, and interact bidirectionally with AD pathology, highlighting the importance of identifying physiological markers for the early detection of AD. Noninvasive approaches for assessing these processes and identifying early biomarkers remain limited. Cerebrospinal fluid (CSF) plays a central role in clearing neurotoxins from the brain, but current methods for quantifying CSF dynamics are invasive, costly, and not well suited for early detection of AD.

**Methods:**

Broadband near-infrared spectroscopy (bbNIRS) provides a promising alternative by enabling noninvasive measurement of total hemoglobin concentration (HbT) and CSF-related free-water fluctuations in the prefrontal cortex (PFC). In this study, we quantified cerebrovascular–CSF coupling using two-channel bbNIRS (2bbNIRS) in three groups: healthy young adults (YA; *n* = 26), healthy older adults (OA; *n* = 27), and early-stage AD patients (*n* = 16). Time series data of 7-min Δ[HbT] and CSF-related Δ[H_2_O]_free_ dynamics were extracted and analyzed within three distinct infraslow oscillatory frequency (endogenic, neurogenic, and myogenic) bands. Linear correlation coefficients and slopes between Δ[HbT] and Δ[H_2_O]_free_ signals were computed to quantify HbT-CSF coupling at each frequency band for each of the three groups, followed by statistical comparisons after Fisher transformation and ANOVA with Bonferroni correction.

**Results:**

The results showed that (1) HbT-CSF coupling was significantly stronger in the AD group than in healthy OA across all frequency bands, and (2) coupling exhibited clear age dependence, with YA showing the weakest prefrontal HbT-CSF coupling.

**Discussion:**

These trends may reflect age- and disease-related reductions in cerebrovascular elasticity and intracranial compliance. Enhanced coupling in the AD group may represent an early compensatory response to impaired CSF transport. Overall, this work demonstrates that 2bbNIRS offers a noninvasive, low-cost method for quantifying cerebrovascular-CSF interactions in the human PFC, with promising potential as a physiological marker of brain aging and early AD.

## 1 Introduction

Alzheimer’s disease (AD) is the most common form of dementia involving cognitive decline. The brain changes of Alzheimer’s disease include excessive accumulation of a protein called beta-amyloid and protein tau. These are responsible for the damage and destruction of neurons (Alzheimers Dementia, 2024). AD currently affects an estimated 55 million people worldwide as of 2020, and this number is projected to increase to approximately 78 million by 2030 (Velandia et al., 2022). This is the reason that early detection is necessary to provide early intervention to reduce or minimize the growing population of patients with severe AD. The advancements in PET, whole exome sequencing, and CSF biomarkers are either very expensive or invasive. Hence there is an ongoing effort for finding ideal biomarkers that are non-invasive, cost effective, easily accessible, and accurate enough to identify the patients with AD at early stages (Georgakas et al., 2023; Mattsson et al., 2023). Numerous studies have employed noninvasive and cost-effective modalities, such as electroencephalography (EEG) and functional near-infrared spectroscopy (fNIRS), to identify potential neurophysiological biomarkers.

Cerebrospinal fluid (CSF) is a critical biomarker source for AD, as CSF contains direct measures of pathological proteins, such as amyloid-β and tau. Quantifying these analytes provides sensitive insight into underlying neurodegenerative processes and supports more accurate diagnostic assessment (Papaliagkas et al., 2023). However, these studies are invasive and not easily accessible. Normally, among the fluids in the body, CSF, which consists of approximately 99% water, is produced and replenished several times daily. CSF plays a vital role in sustaining brain health (Spector et al., 2015) because its circulation acts as a cleansing mechanism for the interstitial fluid of the brain and removes neurotoxic waste products, including degraded proteins and metabolites. This process is part of the glymphatic drainage system, which is a network of spaces in the brain that removes waste and helps distribute other compounds. In particular, the glymphatic drainage system eliminates the accumulation of toxins (Dreha-Kulaczewski et al., 2015), such as amyloid-β (Aβ), which is a precursor to neuronal dysfunction. This waste-clearance process is significantly enhanced during sleep because of increased CSF flow (Nedergaard, 2013; Semyachkina-Glushkovskaya et al., 2023).

**FIGURE 1:**
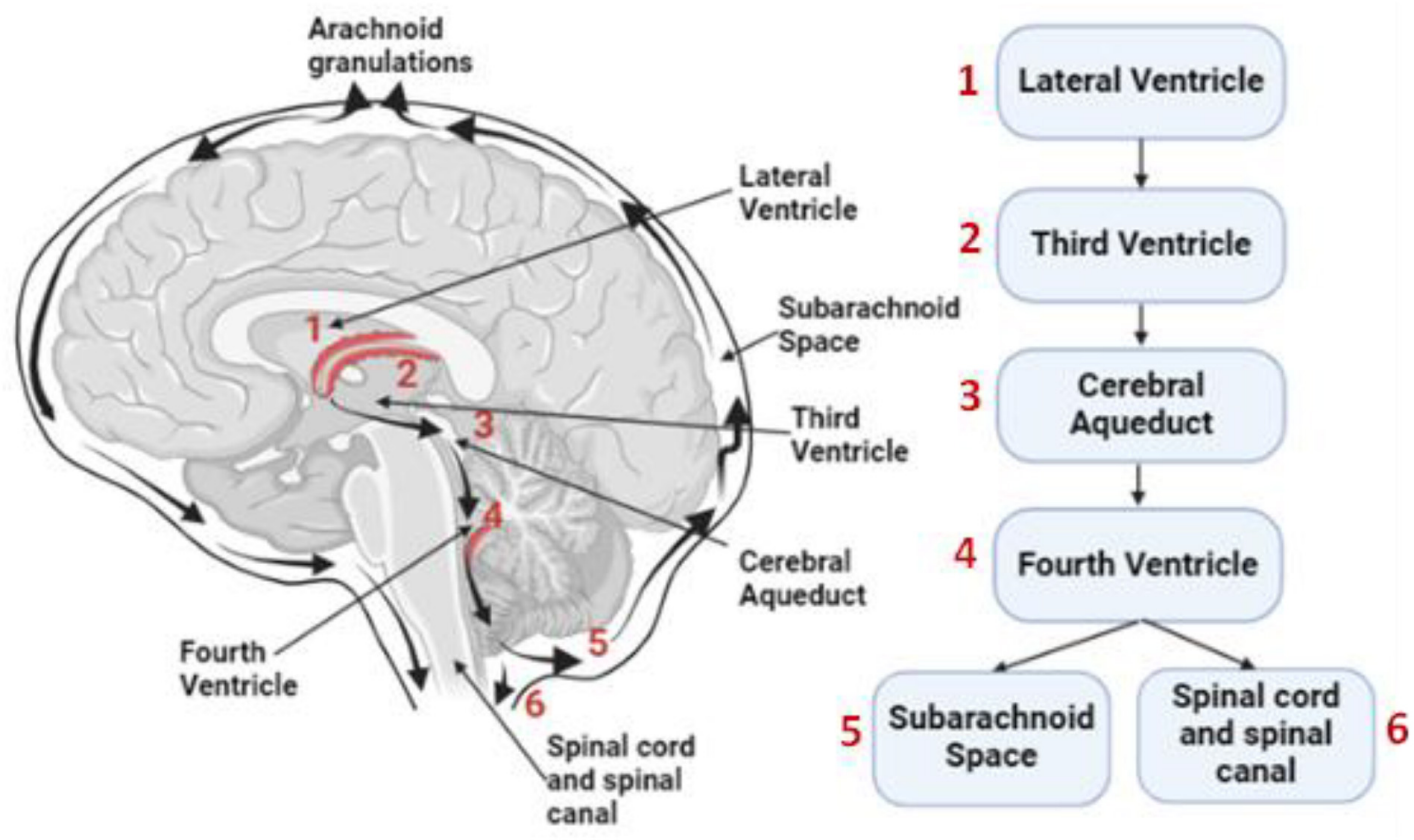
Illustration of CSF flow in the human brain. CSF circulation at the macroscopic level begins in the lateral ventricles (1), passes through the third (2) and fourth (4) ventricles via the cerebral aqueduct (3), and then flows into the subarachnoid space (5) and spinal canal (6). Arrows indicate the principal CSF flow directions.

Figure 1 depicts the macroscopic view of CSF circulation. Given the key role played by the glymphatic system in human brain health, it is highly desirable to noninvasively monitor and quantify CSF dynamics in patients with neurological diseases and in healthy older adults (OA) wishing for healthy longevity.

Prior literature suggests that CSF may serve as a biomarker of brain aging, as its composition, flow, and clearance dynamics change with age. For example, Yamada et al. (2023) reported that CSF from the “young” brain can exert restorative effects on the aging brain, whereas advancing age is linked to reduced CSF flow, impaired metabolic waste clearance, and cognitive decline accompanied by gray-matter atrophy. Moreover, age-dependent neurological disorders, such as dementia, are closely associated with CSF dynamic and drainage disruption. Thus, quantifying alterations in coupling between cerebral total hemoglobin concentration (HbT) and free water content in CSF (H_2_O_*free*_), which represents cerebrovascular-CSF coupling, offers an indirect but valuable means to detect weakening or/and early impairment in CSF dynamics.

Conventional or near-infrared spectroscopy (NIRS) with a 3-cm source–detector separation primarily interrogates superficial head tissues located under the optode pair, including the scalp, skull, subarachnoid CSF, and the superficial cortical gray matter. Due to the limited penetration depth (∼1–1.5 cm), the detected NIR signals predominantly originate from these superficial, subarachnoid, and cortical layers. Consequently, the NIR-derived water content primarily reflects water within the subarachnoid CSF space rather than ventricular or deep subcortical CSF compartments (Myllylä et al., 2018). A novel analysis, introduced recently by Myllylä et al., employed conventional NIRS with 4 wavelengths (660, 830, 740, and 980 nm) to monitor fluctuations in cerebral free water content or CSF, enabling the quantification of CSF dynamics in the prefrontal cortex (Myllylä et al., 2018). Building on this new development, a study employing 4-wavelength NIRS identified an inverse linear correlation between normalized alterations in HbT and H_2_O_*free*_ in 51 healthy participants (Borchardt et al., 2021). This new analysis method provides fresh or additional metrics for assessing brain health in humans with or without neurological disorders, with or without intervention. For example, it can help identify clinically meaningful CSF features in patients with AD, which may serve as potential biomarkers for the early diagnosis of AD.

This study aimed to examine whether the metrics of HbT-CSF coupling in the prefrontal cortex (PFC) measured using two-channel broadband NIRS (2bbNIRS) (Shahdadian et al., 2022; Shahdadian et al., 2023) can serve as non-invasive neurophysiological features for the early detection of AD. Specifically, this investigation aimed to explore the significant differences in the coupling metrics of HbT-CSF between healthy OA and patients with early stage AD for identifiable characteristics of the AD brain. These coupling metrics were also compared with those observed in healthy YA to evaluate the age-dependent alterations in HbT-CSF coupling. By the end of the study, our results suggest that 2bbNIRS offers a noninvasive, low-cost method for quantifying cerebrovascular-CSF interactions in the human PFC, with strong potential as a physiological marker of brain aging and early AD.

## 2 Materials and methods

### 2.1 Study participants

For this study, we recruited 46 OA from the Dallas–Fort Worth community, including 30 cognitively healthy participants and 16 patients with AD (8 males, 8 females; mean age ± SD = 72.8 ± 8.3 years). All 16 AD cases were confirmed based on physician diagnoses and included in the analysis. The inclusion and exclusion criteria for both healthy OA and patients with AD can be found in Supplementary material 1. To examine age-dependent differences, we incorporated data from a previously published study that did not assess CSF-related dynamics (Shahdadian et al., 2022; Shahdadian et al., 2023). In that study, 31 healthy YA were recruited from the University of Texas at Arlington and screened using the same inclusion criteria as those in our prior work (Wang et al., 2016; Wang et al., 2017). Participants completed a protocol involving a 7-min pre-stimulation baseline, 8-min light stimulation, and 7-min post-stimulation period (Shahdadian et al., 2022; Shahdadian et al., 2023), with each individual attending five visits, including two sham sessions. The 7-min pre-stimulation baselines from all five visits were averaged to compute correlation and slope values as one resting-state baseline for each young participant. All study procedures were approved by the Institutional Review Board of the University of Texas at Arlington, and informed consent was obtained from all participants prior to measurement.

Since bbNIRS measurements are sensitive to motion artifacts, several subjects from each group were excluded from the analysis due to excessive noise or incomplete data collection. After such exclusion, 27 out of 30 healthy OA (5 males, 22 females; mean age ± SD = 67 ± 5.6 years), 16 patients with AD, and 26 of 31 healthy YA (14 males, 12 females; mean age ± SD = 22.4 ± 2.3 years) were included for further data analysis.

### 2.2 Experimental setup and protocol for bbNIRS measurements

**FIGURE 2:**
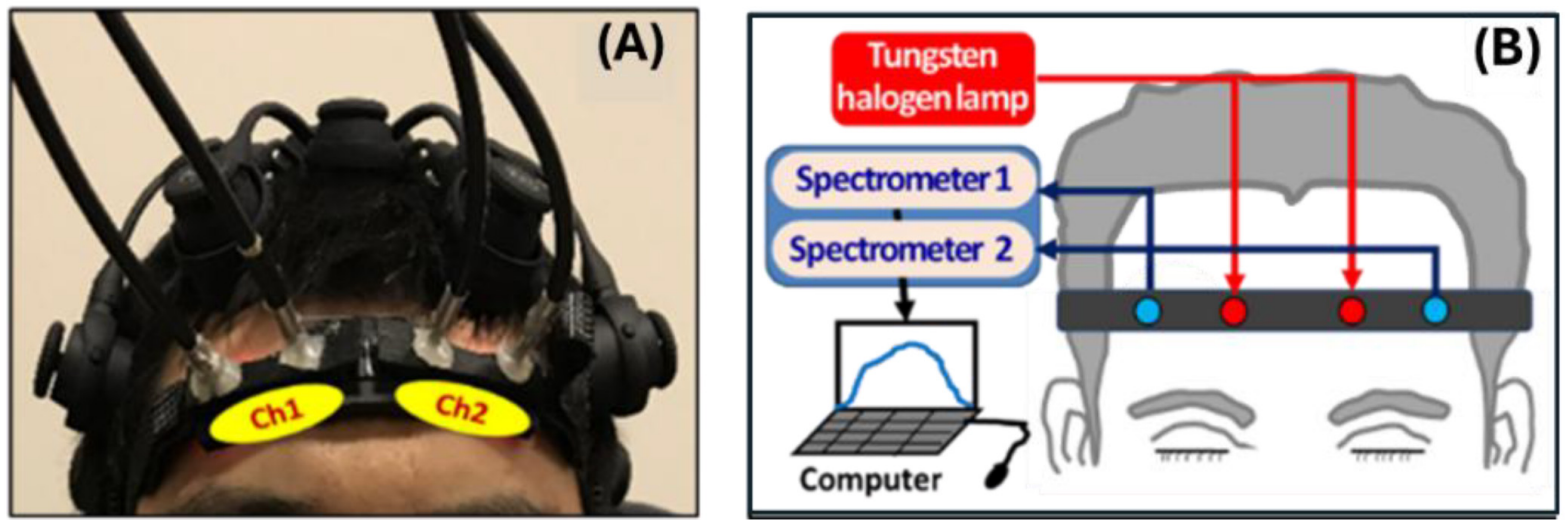
Illustration of the experimental setup. **(A)** A photo graph showing two optical probes of a 2bbNIRS device placed on the forehead of a participant. Multiple legs of an electroencephalogram (EEG) on the head were also attached to the head for concurrent EEG readings, which are not the scope of this paper and are thus not presented hereafter. **(B)** A schematic of the 2bbNIRS setup.

The 2bbNIRS experimental setup is shown with a photo and schematic in Figures 2A,B, respectively, as detailed in Shahdadian et al. (2022). Briefly, the 2-channel system consisted of two branches of a broadband white light source (OSL2, Thorlabs Inc, NJ, NY, United States) and two CCD array spectrometers (QEPro, Ocean Optics Inc., Orlando, FL, United States) with a spectral detection range of 740–1,100 nm. The 2bbNIRS recording channels were positioned symmetrically on the subject’s forehead according to visual judgment. Each channel consisted of one fiber bundle for light delivery to the forehead and another for backscattered light collection from the brain tissue, with a source–detector separation of 3 cm. A 2-channel probe holder was designed and 3D printed with a flexible material to ensure comfortable and firm attachment of the fiber bundles to the forehead skin, accommodating each participant’s forehead curvature.

With the setup shown above, resting-state 2bbNIRS data were collected under a 7-min eyes-closed condition from both healthy OA and patients with AD (Shahdadian et al., 2023; Sissons et al., 2023). The participants were seated in a comfortable sofa chair in a sitting position and asked to minimize any movement during the data collection period. Similar equipment setup and resting-state measurement protocol were also performed from a group of YA (Shahdadian et al., 2022).

### 2.3 Data analysis

**FIGURE 3:**
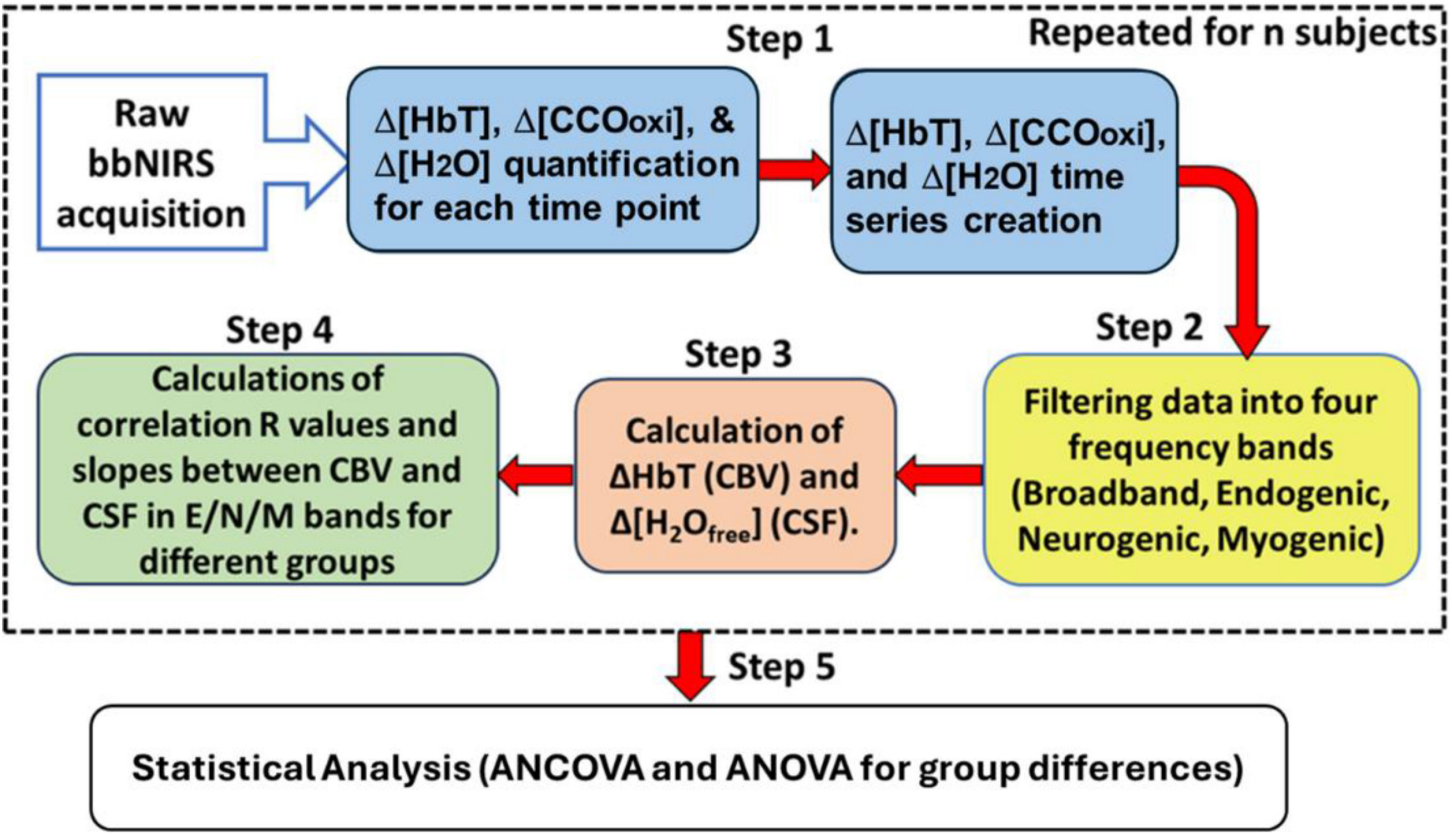
Data processing flowchart with five steps. Step 1: Quantify Δ[HbO] or Δ[HbT], Δ[CCOoxi], and Δ[H_2_O] at each time point, followed by the construction of time series for each metric or variable (blue boxes). Step 2: Filter each time series, Δ[HbT], Δ[CCOoxi], and Δ[H_2_O], into four infraslow frequency ranges in the broadband, endogenic (E), neurogenic (N), and myogenic (M) bands (yellow box). Step 3: Calculate the frequency-specific time series of Δ[HbT] (representing CBV) and Δ[H_2_O]_*free*_ (representing CSF); the latter is obtained by subtracting the normalized Δ[HbT] from the normalized Δ[H_2_O]_*NIRS*_ (orange box). Step 4: Determine the linear correlation coefficients and slopes between Δ[HbT] and Δ[H_2_O]_*free*_ (or Δ[H_2_O]_*CSF*_) in each E/N/M band (green box) with the averaged data between both lateral sides of the forehead. The big dashed box outlines the four steps repeated for the respective participants and the respective time periods. Step 5: Statistical testing for significant differences in each neurophysiological parameter in each E/N/M band between healthy OA and patients with AD.

The algorithms used to quantify alterations in oxygenated and deoxygenated hemoglobin concentrations [Δ(HbO) and Δ(HHb), respectively] as well as the oxidized state of cytochrome c oxidase [Δ(CCOoxi)] of living tissue or the human cortex have been published in several studies (Bale et al., 2016; Leadley et al., 2024). The newly developed algorithm used in this study is the addition of calculations of the water content in the PFC, Δ[H_2_O], and the corresponding free water content in the CSF, Δ[H_2_O]_*free*_. Figure 3 is a flowchart outlining the overall data-processing procedure, with steps describing the derivation of the newly added Δ[H_2_O]_*free*_ quantities.

#### 2.3.1 STEP 1: conversion of ΔOD(t, λ) to Δ[HbO](t), Δ[CCOoxi](t) and Δ[H_2_O](t) over time

For bbNIRS data acquisition, the sampling rate was 0.67 Hz (1.5 sec per spectrum), resulting in 280 temporal points during the 7-min measurement period. Each data point represents an NIR spectrum (740–1,100 nm) with a spectral interval of 0.38 nm (Wang et al., 2017; Shahdadian et al., 2022; Shahdadian et al., 2023). The first data processing step was to normalize or calibrate the raw spectral data to the initial measurement spectrum at time *t* = 0, defined in Equation (1) as:


$$\Delta \,O\,D\,\left(t,\lambda \right)=l\,o\,g_{10}\,\left[\frac{I_{0}\left(t=0,\lambda \right)}{I\,\left(t,\lambda \right)}\right],$$ (1)

where *I_0_(t = 0, λ)* is the baseline spectrum at time *t* = 0 or an average of several initial baseline spectral readings, *I(t, λ)* represent the time-resolved NIR spectra acquired at each time point, *t*, throughout the entire experimental duration, and ΔOD(t, λ) is a relative optical density spectrum at time, *t*, and wavelength λ. One benefit of utilizing ΔOD(t, λ) in cerebral tissue quantification is its self-calibration or self-normalization ability with respect to the initial spectral reading at t = 0. This approach minimizes the effects of variations in probe positioning and baseline alterations among participants.

According to the modified Beer-Lambert law, ΔOD(λ) is related to tissue chromophores as written in Equation (2) (Bale et al., 2016; Leadley et al., 2024):


ΔOD(λ)={ε(λ)HbOΔ[HbO]+ε(λ)HHbΔ[HHb]+ (2)

ε(λ)ΔC⁢C⁢OΔ[CCOoxi]+ε(λ)H2⁢OΔ[HO2]}L(λ)

where *ε_*i*_*(λ) is the optical extinction coefficient at λ, and the index *i* includes chromophores of [HbO], [HHb], and [CCOoxi]. However, *ε_Δ*CCO*_*(λ) is the difference extinction coefficient spectrum of cytochrome-c-oxidase, as shown in Equation (3), which is defined as the difference between the extinction coefficients of its oxidized and reduced forms:


$$\varepsilon_{\Delta \,C\,C\,O}\,\left(\lambda \right)=\varepsilon_{o\,x\,i\,d\,i\,z\,e\,d}\,\left(\lambda \right)-\varepsilon_{r\,e\,d\,u\,c\,e\,d}\,\left(\lambda \right).$$ (3)

In Equation 2, ε_H_2O_(λ) represents light absorption of water at λ with a factor of 2.3, namely, ε_H_2O_(λ) = μ_*a*_(λ)/2.3, Δ[H_2_O] is in % of volume (Bigio and Fantini, 2016). Also, L(λ) is the optical pathlength that NIR light travels through the tissue and is equal to a differential pathlength factor (DPF) multiplied by the source-detector separation, r, namely, L(*λ)* = r × DPF(*λ)*. DPF(λ) can be calculated using diffusion theory (Wang et al., 2016; Wang et al., 2017), as detailed in Supplementary material 2. Given a broadband spectrum from *λ_1_ to λ_*n*_*, Equation (2) can be written as follows:


$$\left[\begin{array}{c} \frac{△\,O\,D\,\left(\lambda_{1}\right)}{L\,\left(\lambda_{1}\right)}\\ \begin{array}{c} \frac{△\,O\,D\,\left(\lambda_{2}\right)}{L\,\left(\lambda_{2}\right)}\\ \begin{array}{c} \frac{△\,O\,D\,\left(\lambda_{3}\right)}{L\,\left(\lambda_{3}\right)} \end{array}\\ \ldots \end{array}\\ \frac{△\,O\,D\,\left(\lambda_{n}\right)}{L\,\left(\lambda_{n}\right)} \end{array}\right]=\left[\begin{array}{c} \varepsilon_{H\,b\,O}\,\left(\lambda_{1}\right)\\ \begin{array}{c} \varepsilon_{H\,b\,O}\,\left(\lambda_{2}\right)\\ \begin{array}{c} \varepsilon_{H\,b\,O}\,\left(\lambda_{3}\right) \end{array}\\ \ldots \end{array}\\ \varepsilon_{H\,b\,O}\,\left(\lambda_{n}\right) \end{array}\right]\,△\,\left[H\,b\,O\right]+$$ (4)

$$\left[\begin{array}{c} \varepsilon_{H\,H\,b}\,\left(\lambda_{1}\right)\\ \begin{array}{c} \varepsilon_{H\,H\,b}\,\left(\lambda_{2}\right)\\ \begin{array}{c} \varepsilon_{H\,H\,b}\,\left(\lambda_{3}\right) \end{array}\\ \ldots \end{array}\\ \varepsilon_{H\,H\,b}\,\left(\lambda_{n}\right) \end{array}\right]\,△\,\left[H\,H\,b\right]+\left[\begin{array}{c} \varepsilon_{\Delta \,C\,C\,O}\,\left(\lambda_{1}\right)\\ \begin{array}{c} \varepsilon_{\Delta \,C\,C\,O}\,\left(\lambda_{2}\right)\\ \begin{array}{c} \varepsilon_{\Delta \,C\,C\,O}\,\left(\lambda_{3}\right) \end{array}\\ \ldots \end{array}\\ \varepsilon_{\Delta \,C\,C\,O}\,\left(\lambda_{n}\right) \end{array}\right]\,△\,\left[C\,C\,O_{o\,x\,i}\right]+$$

$$\left[\begin{array}{c} \varepsilon_{H_{2}\,O}\,\left(\lambda_{1}\right)\\ \begin{array}{c} \varepsilon_{H_{2}\,O}\,\left(\lambda_{2}\right)\\ \begin{array}{c} \varepsilon_{H_{2}\,O}\,\left(\lambda_{3}\right) \end{array}\\ \ldots \end{array}\\ \varepsilon_{H_{2}\,O}\,\left(\lambda_{n}\right) \end{array}\right]\,△\,\left[H_{2}\,O\right].$$

After writing Equation (4) in a matrix format and then converting it, we obtain Equation (5), which facilitates quantifications of Δ[HbO], Δ[HHb], Δ[CCOoxi], and Δ[H_2_O] of living tissues under study (Shahdadian et al., 2022; Shahdadian et al., 2023).


$$\left[\begin{array}{c} \Delta \,\left[H\,b\,O\right]\\ \Delta \,\left[H\,H\,b\right]\\ \Delta \,\left[C\,C\,O_{o\,x\,i}\right]\\ \Delta \,\left[H_{2}\,O\right] \end{array}\right]=\frac{1}{r}$$ (5)

$${\left[\begin{array}{c} \begin{array}{ccc} \varepsilon_{H\,b\,O}\,\left(\lambda_{1}\right) & \varepsilon_{H\,H\,b}\,\left(\lambda_{1}\right)\,\varepsilon_{\Delta \,C\,C\,O}\,\left(\lambda_{1}\right) & \varepsilon_{H_{2}\,O}\,\left(\lambda_{1}\right)\\ \vdots & \ddots & \vdots \\ & \cdots & \end{array}\\ \begin{array}{ccc} \varepsilon_{H\,b\,O}\,\left(\lambda_{m}\right) & \varepsilon_{H\,H\,b}\,\left(\lambda_{m}\right)\,\varepsilon_{\Delta \,C\,C\,O}\,\left(\lambda_{m}\right) & \varepsilon_{H_{2}\,O}\,\left(\lambda_{m}\right)\\ \vdots & \ddots & \vdots \\ \varepsilon_{H\,b\,O}\,\left(\lambda_{n}\right) & \cdots & \varepsilon_{H_{2}\,O}\,\left(\lambda_{n}\right) \end{array} \end{array}\right]}^{-1}$$

$$\left[\begin{array}{c} \frac{△\,O\,D\,\left(\lambda_{1}\right)}{D\,P\,F\,\left(\lambda_{1}\right)}\\ \begin{array}{c} \frac{△\,O\,D\,\left(\lambda_{2}\right)}{D\,P\,F\,\left(\lambda_{2}\right)}\\ \begin{array}{c} \frac{△\,O\,D\,\left(\lambda_{3}\right)}{D\,P\,F\,\left(\lambda_{3}\right)} \end{array} \end{array}\\ \frac{△\,O\,D\,\left(\lambda_{n}\right)}{D\,P\,F\,\left(\lambda_{n}\right)} \end{array}\right]$$

A mathematical derivation from a raw NIR spectrum to the quantification of Δ[HbO], Δ[HHb], Δ[CCOoxi], and Δ[H_2_O] is represented graphically in Supplementary Figure 1, with detailed explanations. After repeating the calculations for each time point, we obtained the time series of Δ[HbO](t), Δ[HHb](t), Δ[CCOoxi](t), and Δ[H_2_O](t) from ΔOD(t, λ).

#### 2.3.2 STEP 2: time series of Δ[HbO], Δ[HbT], Δ[CCOoxi], and Δ[H_2_O] in the E/N/M bands

The time series for Δ[HbO], Δ[HbT], Δ[CCOoxi], and Δ[H_2_O] were subsequently filtered to isolate three distinct frequency bands in accordance with the infraslow oscillation (ISO) standards, referred to as the E/N/M components, as reported (Shahdadian et al., 2022; Wang et al., 2022) and used in previous studies (Shahdadian et al., 2023; Sissons et al., 2023; Shahdadian et al., 2024). Band-pass filtering was performed using the command of “filtfilt” in MATLAB with the zero-phase 10th-order Butterworth filter, which is implemented with forward–reverse filtering to prevent the phase distortion. The 10th-order filter was selected to achieve a steeper roll-off or sharper band separation between the three frequency bands. The frequency ranges used for filtering the E/N/M bands were 0.005–0.02 Hz (endogenic; E), 0.0205–0.039 Hz (neurogenic; N), and 0.04–0.15 Hz (myogenic; M), consistent with prior ISO-based studies. Supplementary Figure 2 graphically illustrates the process of decomposing a Δ[HbO] time series into three frequency bands as an example.

#### 2.3.3 STEP 3: dynamics of the free water content in the CSF

Broadband NIRS with a 3-cm source-detector separation primarily interrogates superficial head tissues, including scalp, skull, subarachnoid CSF, and the superficial cortical gray matter beneath the optode pair. Physiologically, the water signal, measured by bbNIRS, comes mainly from vascular, cerebral tissue, and CSF compartments. Because of the limited penetration depth (∼1–1.5 cm), the measured NIR-derived water signal results from the subarachnoid free water rather than ventricular or deep subcortical CSF compartments (Myllylä et al., 2018). Water within the vasculature and cerebral space are bound by blood vessels and cerebral volumes, respectively, while water in CSF within the subarachnoid space is relatively free to move. Accordingly, we can write the following Equation:


Δ[H2O](t)NIRS=Δ[H2O](t)blood+Δ[H2O](t)CSF+ (6)

Δ[H2O](t)cerebral⁢_⁢tissue.

In principle, the water content within the cerebral volume is not expected to change systemically over a short period (e.g., 7 min) in the resting state of the PFC. Thus, temporal variations in Δ[H_2_O]_*NIRS*_(t) primarily reflect fluctuations in Δ[H_2_O]_*blood*_(t) and Δ[H_2_O]_*CSF*_(t) (Myllylä et al., 2018; Borchardt et al., 2021). Note that Δ[H_2_O]_*CSF*_(t) represents freely mobile water within the subarachnoid space, and Δ[H_2_O]_*blood*_(t) is carried within the vasculature and thus oscillates with Δ[HbT]. Because Δ[HbT] is proportional to CBV, dynamic changes in Δ[HbT] reflect alterations in blood-bound water content. Thus, Δ[H_2_O]_*blood*_(t) is equivalent to Δ[H_2_O]_*HbT*_(t), the latter of which can be included in the measured Δ[H_2_O]_*NIRS*_(t) signal from bbNIRS. Accordingly, Equation (6) becomes


Δ[H2O](t)NIRS=Δ[H2O](t)HbT+Δ[H2O](t)CSF, (7a)

which leads to


Δ[H2O](t)CSF=Δ[H2O](t)NIRS-Δ[H2O](t)HbT, (7b)

where Δ[H_2_O]_*NIRS*_(t) is one of the four recovered metrics from Equation (5), and Δ[H_2_O]_*HbT*_(t) represents temporal fluctuations of blood-bound water within the cerebrovascular space.

The key focus of this study is to examine the dynamic relationship, or coupling, between temporal fluctuations of blood-bound water and free water. Because ΔHbT reflects cerebrovascular blood-volume fluctuations, it can serve as a dynamic surrogate for blood-bound vascular water, expressed as *dy*{ΔHbT} = *dy*{Δ[H_2_O]_*HbT*_(t)}, where dy means “dynamic”. Similarly, temporal variations in Δ[H_2_O]_*CSF*_(t) and Δ[H_2_O]_*NIRS*_(t) can be represented as *dy*{Δ[H_2_O]_*CSF*_(t)} and *dy*{Δ[H_2_O]_*NIRS*_(t)}, respectively. Accordingly, Equation (7b) can be rewritten as


dy{Δ[H2O](t)CSF}=dy{Δ[H2O](t)NIRS}- (8a)

dy{Δ[H2O](t)HbT)}=dy{Δ[H2O](t)NIRS}-dy{Δ[HbT](t))},

where “{Y(t)}” in the equation symbolizes each time series of signals, Y, as a function of time (t), the prefix of “*dy*” indicates “dynamic alteration” for each temporal quantity. Note that we replaced *dy*{Δ[H_2_O]_*HbT*_(t))} by *dy*{Δ[HbT](t))} in Equation (8a) as vascular water is expected to exhibit temporal dynamics closely similar to Δ[HbT], which serves as a surrogate for dynamic changes in cerebral blood volume.

Because hemoglobin absorbs NIR light more strongly than water in the 780–900 nm range, the measured amplitude of ΔHbT is substantially larger than that of ΔH_2_O [based on Equation (5)], such that ΔHbT(t) is much larger than Δ[H_2_O](t) (and thus than Δ[H_2_O]_*CSF*_(t)). However, Equation (8) characterizes dynamic variations rather than absolute values of these three metrics. Therefore, normalization (between −1 and 1) for each of the three cerebral dynamic parameters was performed to extract their dynamic characteristics (Myllylä et al., 2018; Borchardt et al., 2021). Under this normalization strategy, Equation (8a) reduces to Equation (8b), enabling isolation of free-water fluctuations associated with CSF dynamics.


{Δ[H2O](t)CSF}nor={Δ[H2O](t)NIRS}nor-{Δ[HbT](t))}nor, (8b)

where the subscript of “nor” indicates “normalized” for each temporal quantity. Note that we replaced {Δ[H_2_O]_*HbT*_(t))}_*nor*_ by {Δ[HbT](t))}_*nor*_ in Equation (8b) as vascular water is expected to exhibit temporal dynamics closely similar to Δ[HbT]. Since Δ[H_2_O]_*NIRS*_(t) and Δ[HbT](t) are obtained from Equation (5) using bbNIRS measurements, the dynamic alterations of Δ[HbT](t) and Δ[H_2_O]_*CSF*_(t) can be derived, enabling quantification of their coupling.

#### 2.3.4 STEP 4: linear fitting of correlation coefficients and slopes between CBV and CSF

To investigate cerebrovascular-CSF coupling in the human PFC, the time series of Δ[HbT] (as CBV) and Δ[H_2_O]_*free*_ (as CSF) from each lateral measurement of the forehead were linearly fitted to obtain Pearson’s correlation coefficient, R, and the slope between Δ[HbT] and Δ[H_2_O]_*free*_ in each frequency E/N/M band per participant. Pearson’s correlation coefficient (R) reflects the strength of the temporal association between the two signals. Since the slope is obtained by fitting a least-squares linear regression between Δ[HbT] and Δ[H_2_O]_*free*_, it indicates the sensitivity of Δ[H_2_O]_*free*_ to Δ[HbT]. The correlation coefficients (R) and slope values from the two lateral probes were averaged to produce a single set of R and slope metrics for each participant in each E/N/M band during the 7-min 2bbNIRS recording. Both R and slope values for the coupling between Δ[HbT(t)] and Δ[H_2_O(t)]_*free*_ are expected to be negative, consistent with the Monro–Kellie hypothesis, which states that the combined volume of brain tissue, intracranial blood, and CSF remains constant (Myllylä et al., 2018; Borchardt et al., 2021). Both the fitted correlation coefficients and slopes in each of the three frequency bands were obtained at the group level and compared against their appropriate counter groups.

#### 2.3.5 STEP 5: statistical analysis

We applied the Fisher z-transformation to all correlation coefficients to improve normality across the three participant groups prior to statistical testing. First, to determine whether physiological parameters (e.g., cerebrovascular-CSF coupling) differed between OA and patients with AD independent of age, an analysis of covariance (ANCOVA) was conducted with group (OA vs. AD) as the fixed factor and age as a continuous covariate. The group × age interaction term was initially tested to evaluate the homogeneity-of-R (and slopes) assumption. As the interaction was not significant, it was removed from the final model. Adjusted group differences were estimated from the model coefficients. Effect sizes were computed using Cohen’s d, with thresholds of 0.2–0.5, 0.5–0.8, 0.8–1.3, and > 1.3 indicating small, medium, large, and very large effects, respectively.

Next, for three-group comparisons involving YA, OA, and AD patients, we analyzed the data using one way analysis of variance (ANOVA) within general linear model framework, followed by planned pairwise contrasts between groups with significance defined as *p* < 0.05. To control multiple comparisons across three groups, we applied Bonferroni correction. The resulting adjusted significance thresholds were *p* < 0.017 (0.05/3), *p* < 0.003 (0.01/3), and *p* < 0.0003 (0.001/3) for the correlation coefficient (*R*) and slope metrics.

## 3 Results

### 3.1 Correlations of HbT and CSF in the PFC of OA with and without AD

**FIGURE 4:**
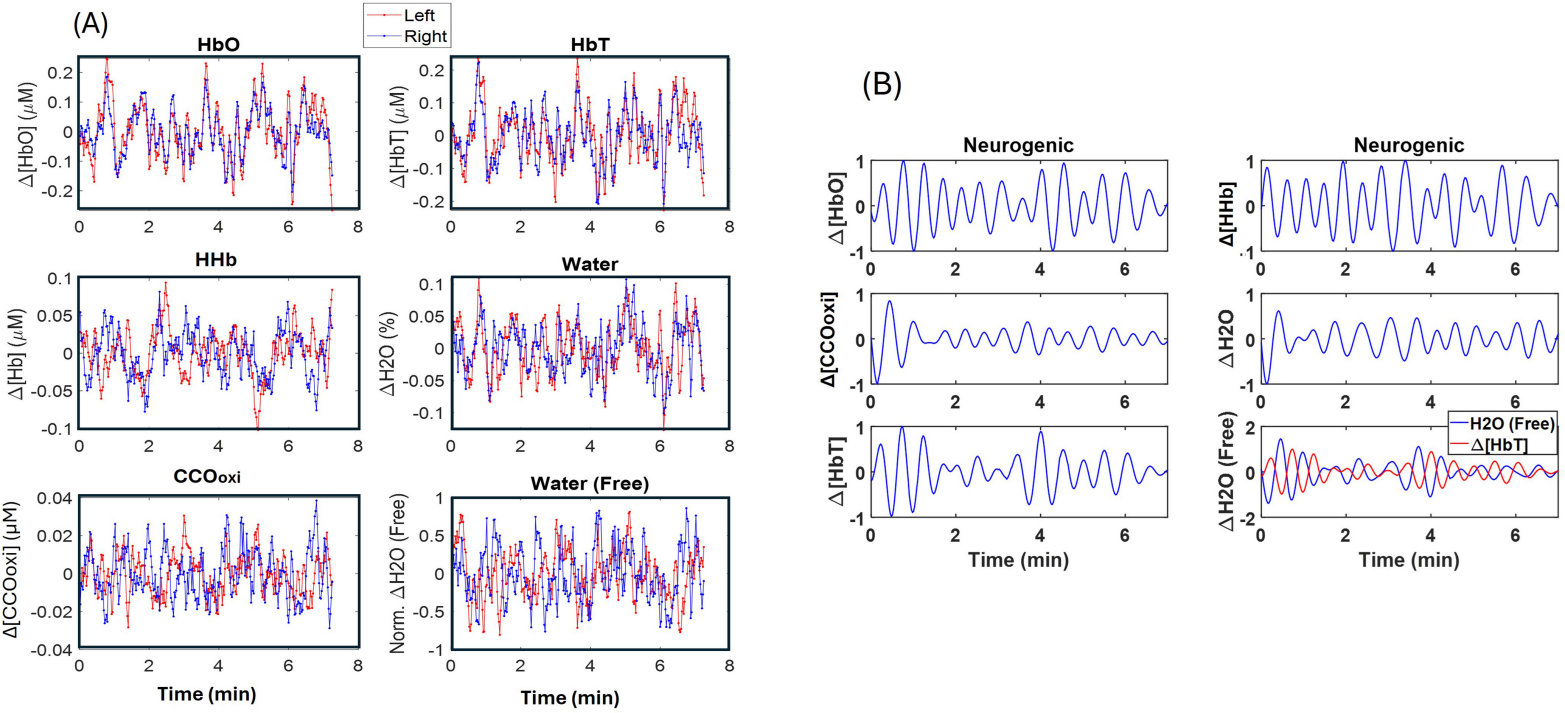
Time series of six neurophysiological parameters from a single participant. **(A)** An example of a set of time series of Δ[HbO], Δ[HHb], Δ[HbT], Δ[CCOoxi], Δ[H_2_O], and normalized Δ[H_2_O]_*free*_ from a participant. Two temporal files in each subpanel show the respective readings from the left (red) and right (blue) PFC of the participant. **(B)** An example of normalized time series of Δ[HbO], Δ[HHb], Δ[HbT], Δ[CCOoxi], Δ[H_2_O], and Δ[H_2_O]_*free*_ (blue) in the neurogenic band. Note that the Δ[H_2_O]_*free*_ time series is plotted together with the normalized Δ[HbT] (red), showing anti-correlated patterns between them.

Following the methodology described in section 2, we quantified temporal alterations in (1) Δ[HbO], (2) Δ[HHb], (3) Δ[HbT] ( = Δ[HbO] + Δ[HHb]), (4) Δ[CCOoxi], (5) total water content measured by NIRS in the PFC (Δ[H_2_O]_*NIRS*_), and (6) free dynamic water content in the CSF (Δ[H_2_O]_*free*_) from each channel. Figure 4A shows such a set of time series for all six quantities. Furthermore, all six time series were normalized between −1 and 1 after being decomposed into three frequency bands of infraslow oscillation (Shahdadian et al., 2022; Wang et al., 2022), namely, E (0.005–0.02 Hz), N (0.02–0.04 Hz), and M (0.04–0.1 Hz) frequencies. As examples, Figure 4B shows a set of time series in the neurogenic band for each of the six neurophysiological quantities. The subpanel on the bottom right of Figure 4B shows temporal profiles of normalized Δ[HbT] (red) and Δ[H_2_O]_*free*_ (blue), exhibiting inverse oscillation patterns between them. The time series data of all three E/N/M bands can be found in Supplementary Figure 3.

**FIGURE 5:**
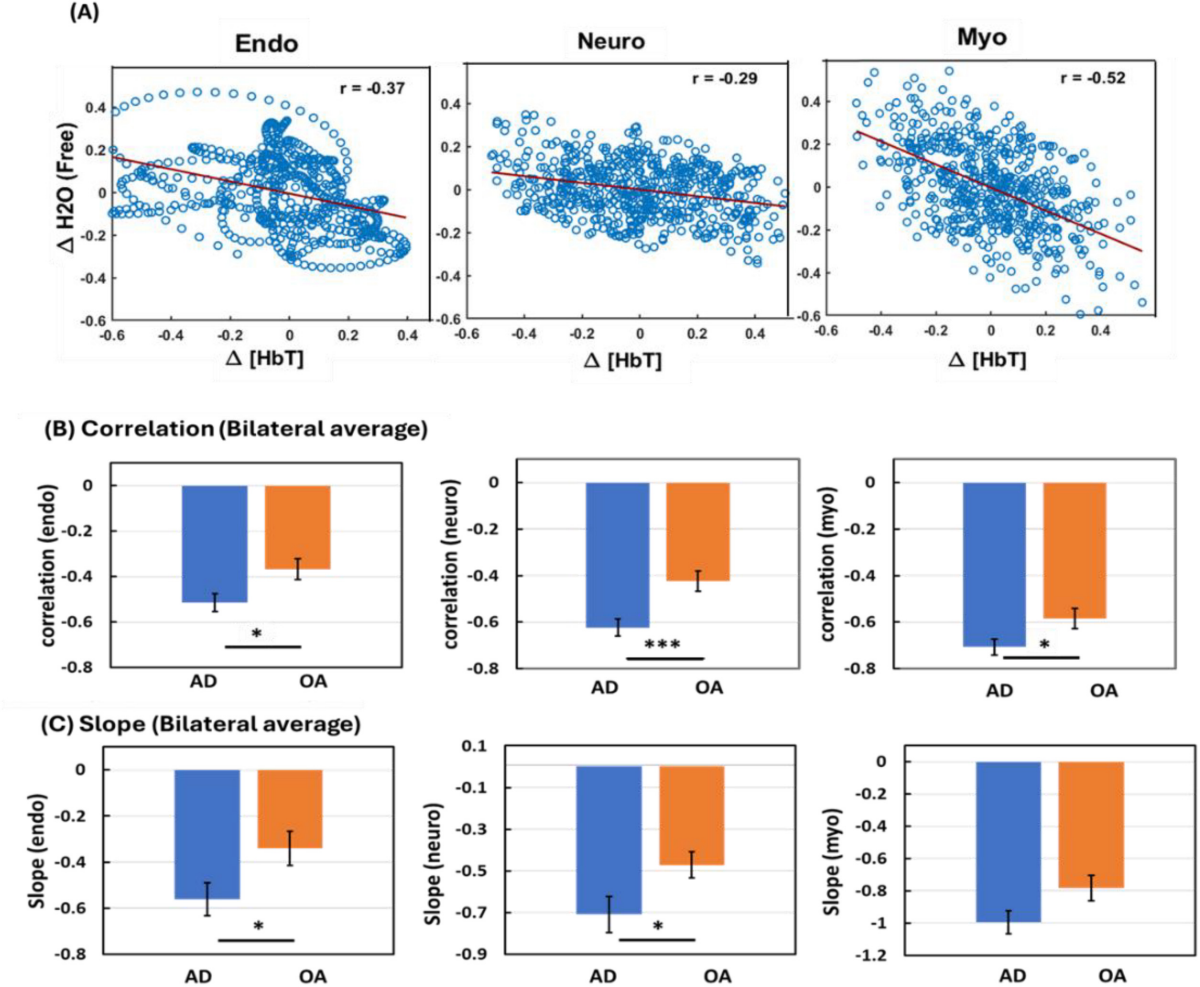
Comparisons of HbT-CSF coupling between AD patients and healthy OA. **(A)** An example of relationships between normalized Δ[HbT] and Δ[H_2_O]_*free*_ with linear correlation fitting in each of the three E/N/M bands from an older adult. Meanwhile, the slopes were calculated by performing least-squares linear regression on the paired time-series data for each frequency band. The “r” values labeled in the three panels are Pearson’s correlation coefficients. **(B)** The correlation coefficients (R) and **(C)** correlation slope values of bilaterally averaged HbT-CSF coupling in the PFC of AD patients (*n* = 16) and healthy OA (*n* = 27) in three frequency bands: the endogenic (0.005–0.02 Hz), neurogenic (0.02–0.04 Hz), and myogenic (0.04–0.1 Hz) bands. Error bars were obtained from the SEM of each respective group. *p < 0.05, ***p < 0.001 by ANCOVA after Fisher transformation.

Given the inverse oscillation patterns between normalized Δ[HbT] and Δ[H_2_O]_*free*_, linear correlation fitting was performed to assess HbT-CSF coupling in each of the three E/N/M bands (Figure 5A) for each lateral side of the bbNIRS measurements. This analysis yielded two correlation metrics for each oscillation band: (1) the correlation coefficient and (2) the correlation slope. To evaluate HbT-CSF coupling metrics at the group level, the same fitting process was applied to both PFC sides for the two subgroups: healthy OA (*n* = 27) and patients with AD (*n* = 16). By averaging the metrics across the two lateral channels within each subgroup, we obtained group-level correlation coefficients (Figure 5B) and slopes (Figure 5C) in each of the E/N/M bands of the PFC.

For comparisons between OA and AD, group differences were evaluated using a multiple linear regression model or ANCOVA following Fisher z-transformation of correlation coefficients. The statistical analysis results revealed that the correlation coefficients *R* (Table 1) of HbT-CSF coupling in the PFC of AD patients were significantly more negative than those of healthy OA in all E/N/M oscillation bands with medium to large effect sizes, as indicated by Cohen’s *d* (−0.68, –1.17, and −0.70, respectively; Table 1). Similarly, the correlation slopes of the AD patients were significantly more negative than those of healthy OA in the E/N bands and marginally significant (*p* = 0.053) in the M band. Age was also included as a covariate in the ANCOVA model; however, neither the effect of age nor the Age × Group interaction (all *p* > 0.05) was significant, indicating that the coupling strength was not driven by age differences between OA and AD groups. Effect sizes were between −0.63 and −0.85 across the three bands (−0.70, −0.85, and −0.63, respectively; Table 2).

**TABLE 1:** Statistical results (based on ANCOVA) of bilaterally averaged correlation coefficients between healthy OA (*n* = 27) and AD patients (*n* = 16).

Frequency	Endogenic (0.005–0.02 Hz)	Neurogenic (0.02–0.04 Hz)	Myogenic (0.04–0.1 Hz)
*p*-value (correlation coefficient)	0.038	< 0.001	0.031
Cohen’s D	−0.68	−1.17	−0.70

**TABLE 2:** Statistical results (based on ANCOVA) of bilaterally averaged slope values between healthy OA (*n* = 27) and AD patients (*n* = 16).

Frequency	Endogenic (0.005–0.02 Hz)	Neurogenic (0.02–0.04 Hz)	Myogenic (0.04–0.1 Hz)
*p-*value (correlation slope)	0.031	0.011	0.053
Cohen’s D	−0.70	−0.85	−0.63

### 3.2 Comparisons of HbT–CSF coupling among young and older adults

**FIGURE 6:**
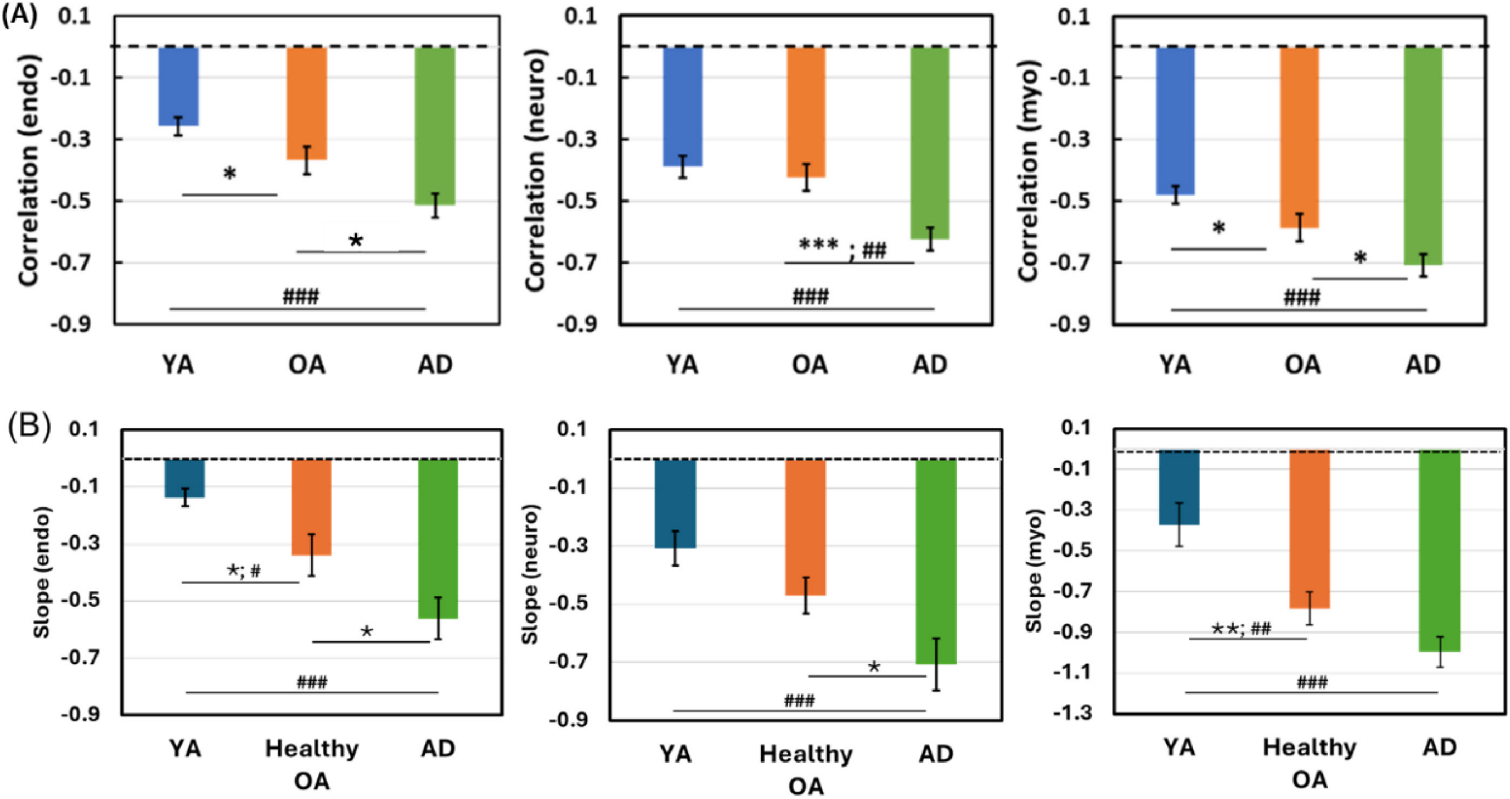
Comparisons of correlation coefficients and slopes in HbT-CSF coupling across different age groups. **(A)** Correlation coefficients and **(B)** correlation slopes of HbT-CSF coupling in the PFC of healthy YA (*n* = 26; blue), healthy OA (*n* = 27; orange), and AD patients (*n* = 16) in three frequency bands: the endogenic (E; 0.005–0.02 Hz; left column), neurogenic (N; 0.02–0.04 Hz; middle column), and myogenic (M; 0.04–0.1 Hz; right column) bands. The error bars represent the SEM for each group. Statistical significance was determined using one-way analysis of variance (ANOVA) followed by planned pairwise contrasts within a general linear modeling framework. *p < 0.05, **p < 0.01, and ***p < 0.001. For multiple comparisons, Bonferroni correction was performed, adjusting the significance thresholds to *p* < 0.0167 (0.05/3), *p* < 0.003 (0.01/3), and *p* < 0.0003 (0.001/3), as marked by #, ##, and ###, respectively.

Regarding HbT-CSF correlation metrics of YA (*n* = 26), 7-min baseline bbNIRS measurements were used for the correlation quantification because such pre-stimulation time series data should be equivalent to those taken from the resting PFC of the other two groups. Thus, we pooled and averaged the respective correlation R and slope values of YA over the five separate baseline measurements and plotted the derived metrics in Figures 6A,B along with those from OA with and without AD for clear comparisons.

Statistical significance was determined using one-way analysis of variance (ANOVA) followed by planned pairwise contrasts within a general linear modeling framework. Additionally, to control multiple comparisons, we applied the Bonferroni correction, adjusting the significance thresholds accordingly. Figure 6 clearly demonstrates progressively increasing trends in both R and slope values from YA to healthy OA and then to patients with AD across all three infraslow E/N/M frequency bands. When considering only the healthy groups, age-related increases in HbT–CSF coupling strength, reflected by both R and slope, were significant in the endogenic and myogenic bands, which are most directly linked to cerebrovascular dynamics. After applying Bonferroni correction to control for multiple comparisons across the three groups, the differences in coupling metrics between YA and patients with AD remained highly significant. Detailed statistical results, including the exact *p*-values for all comparisons, are provided in Supplementary Tables 1, 2.

## 4 Discussion

This study demonstrates the feasibility of quantifying cerebrovascular-CSF coupling in the human prefrontal cortex using advanced analysis of bbNIRS measurements. The key scientific findings include distinct CSF–HbT coupling characteristics in patients with AD compared with OA and YA, and the potential of these coupling metrics to serve as physiological markers for early AD detection. Figure 1 provides a bird’s-eye overview of CSF flow through the ventricular and subarachnoid spaces, though it does not depict the perivascular and interstitial exchange pathways of the glymphatic system. While our coupling measures do not directly quantify glymphatic transport, they nonetheless capture CSF-related physiological dynamics that indirectly reflect aspects of intracranial compliance and CSF regulation, as further discussed in the following sections.

### 4.1 Characterization of cerebrovascular-CSF coupling in OA with and without AD

The Monro-Kellie doctrine states that the total intracranial volume, which comprises brain tissue, CSF, and cerebral blood (CBV), remains constant within the rigid cranial vault (Myllylä et al., 2018; Borchardt et al., 2021). Consequently, dynamic changes in CBV and CSF volume are expected to be inversely related, producing negative correlation coefficients, R, as previously reported in Borchardt et al. (2021). Consistent with this principle, our study observed negative R values and negative slopes between Δ[HbT] and CSF dynamics in healthy OA and in patients with AD (Figure 5). Moreover, cerebrovascular-CSF coupling was significantly tighter in the AD group, with consistently stronger anticorrelations and steeper slopes across all E/N/M frequency bands than its counterpart.

In this context, the Monro-Kellie framework serves as a macro-scale heuristic for interpreting the observed anticorrelation between Δ[HbT] and Δ[H_2_O]_*free*_. This relationship most plausibly reflects large-scale volumetric compensation between vascular and CSF compartments rather than direct evidence of perivascular or glymphatic transport. More specifically, our findings revealed that the cerebrovascular-CSF coupling metrics in early AD are significantly increased. This observation can be attributed to several factors. One of them is associated with AD-related structural changes in the human brain, including overall brain atrophy, increased CSF volume, and reduced CBV (Iliff et al., 2012; Tarumi and Zhang, 2014). Second, the loss of intracranial compliance in patients with AD means that even small changes in the CBV might cause larger changes in CSF oscillations, strengthening the relationship. Finally, the overall conditions of AD, such as vascular stiffness, amyloid-related disruption of neurovascular function, and glymphatic impairment, could amplify the effects of changes in CBV on CSF oscillations.

Cardiac-driven, high-frequency pulsatile dynamics are common sources to drive intracranial compliance. However, our findings should not be interpreted as direct evidence of altered pulsatile compliance because our analysis focuses exclusively on infraslow oscillations (0.005–0.1 Hz). The strengthened HbT-CSF coupling in AD more likely reflects alterations in slow regulatory interactions between cerebral blood volume fluctuations and CSF control mechanisms. These may include modified CSF redistribution within subarachnoid and perivascular spaces, changes in low-frequency pressure-volume buffering capacity, or alterations in CSF production-reabsorption balance.

Moreover, disruptions in CBV-CSF coupling may be indicative of impaired glymphatic function, a hallmark of AD pathology in which the clearance of metabolic waste products is reduced or diminished (Zlokovic, 2011). The consistency of this effect across frequency bands indicates that the alteration is not confined to a single regulatory mechanism but may represent a broader shift in vascular-CSF interactions. Further studies are needed to determine whether this strengthened anti-coupling reflects compensatory adaptation or pathological rigidification of intracranial dynamics.

Our findings align with those of prior studies, suggesting that AD progression is accompanied by impaired cerebrovascular function and reduced glymphatic clearance efficiency (Iadecola, 2004). In addition, we expect that a greater negative correlation and/or steeper slope of CBV–CSF coupling might occur only at the early stage of AD, as the brain tries to compensate for emerging dysfunctions. When the brain approaches the late stage of AD, a weaker correlation and shallower slope are expected because of the widespread breakdown of regulatory mechanisms. Thus, a tighter *R* value and steeper negative slope could indicate early compensatory responses or local dysfunction in the AD brain. This information might provide insight into disease staging.

### 4.2 Characterization of cerebrovascular-CSF coupling as brain ages

Figure 6 illustrates an age-dependent increase in the negative correlations (both R and slope) between the cerebral Δ[HbT] and CSF. This observation can be explained by the following physiological mechanisms. Normal aging is associated with progressive cerebral atrophy, which reduces the parenchymal and cerebral blood volumes. Because the intracranial cavity is rigid, this loss of tissue volume is partially compensated for by the expansion of CSF space. As a result, age-related reductions in Δ[HbT] tend to co-occur with larger fluctuations in CSF volume, thereby amplifying the anti-correlation between them. The combination of a decrease in Δ[HbT] and an increase in CSF volume resulted in a more pronounced inverse relationship between them. Thus, our results provide evidence that both the correlation R values and slopes between Δ[HbT] and CSF may serve as new or supplementary features or markers to characterize brain health and aging.

### 4.3 Underlying mechanism of increased HbT-CSF coupling from YA to OA and then to AD

Given the significant increase in inverse cerebrovascular–CSF coupling observed from YA to OA, and further to patients with AD, we further quantify the underlying physiological mechanism in terms of vascular compliance and intracranial volume compensation. Under the Monro–Kellie doctrine, the total intracranial volume remains approximately constant, such that


$$\Delta \,V_{\text{blood}}+\Delta \,V_{\text{CSF}}+\Delta \,V_{\text{other}}\approx 0$$ (9)

where Δ*V*_blood_ (in proportion to Δ*V*_HbT_) and Δ*V*_CSF_ are the volume alterations of blood and CSF within the PFC interrogated by NIRS. Equation (9) can be reduced to Equation (10) when other tissue-volume changes are negligible.


$$\Delta \,V_{\text{CSF}}\approx -\Delta \,V_{\text{blood}}.$$ (10)

However, the strength of this inverse relationship depends on vascular compliance. In compliant vessels, part of the oscillatory perturbation is buffered by vessel elasticity, so only a fraction of the oscillatory blood-volume change is transmitted to the surrounding intracranial space, as expressed in Equation (11):


$$\Delta \,V_{\text{transmitted}}=\alpha \,\Delta \,V_{\text{blood}},0<\alpha <1,$$ (11)

where αrepresents the fraction of blood-volume oscillation transmitted to drive CSF displacement. Higher compliance of blood vessels in YA results in smaller α and weaker HbT-CSF coupling. In contrast, reduced vascular compliance in OA leads to less buffering and a larger transmitted fraction (α_OA_>α_YA_), thus producing stronger inverse coupling. Accordingly, a larger fraction of Δ*V*_blood_ (i.e., a larger α) is transmitted outward to the intracranial compartment, yielding


$$\Delta \,V_{\text{CSF}}\approx -\alpha \,\Delta \,V_{\text{blood}},\alpha_{\text{OA}}>\alpha_{\text{YA}}.$$ (12)

**FIGURE 7:**
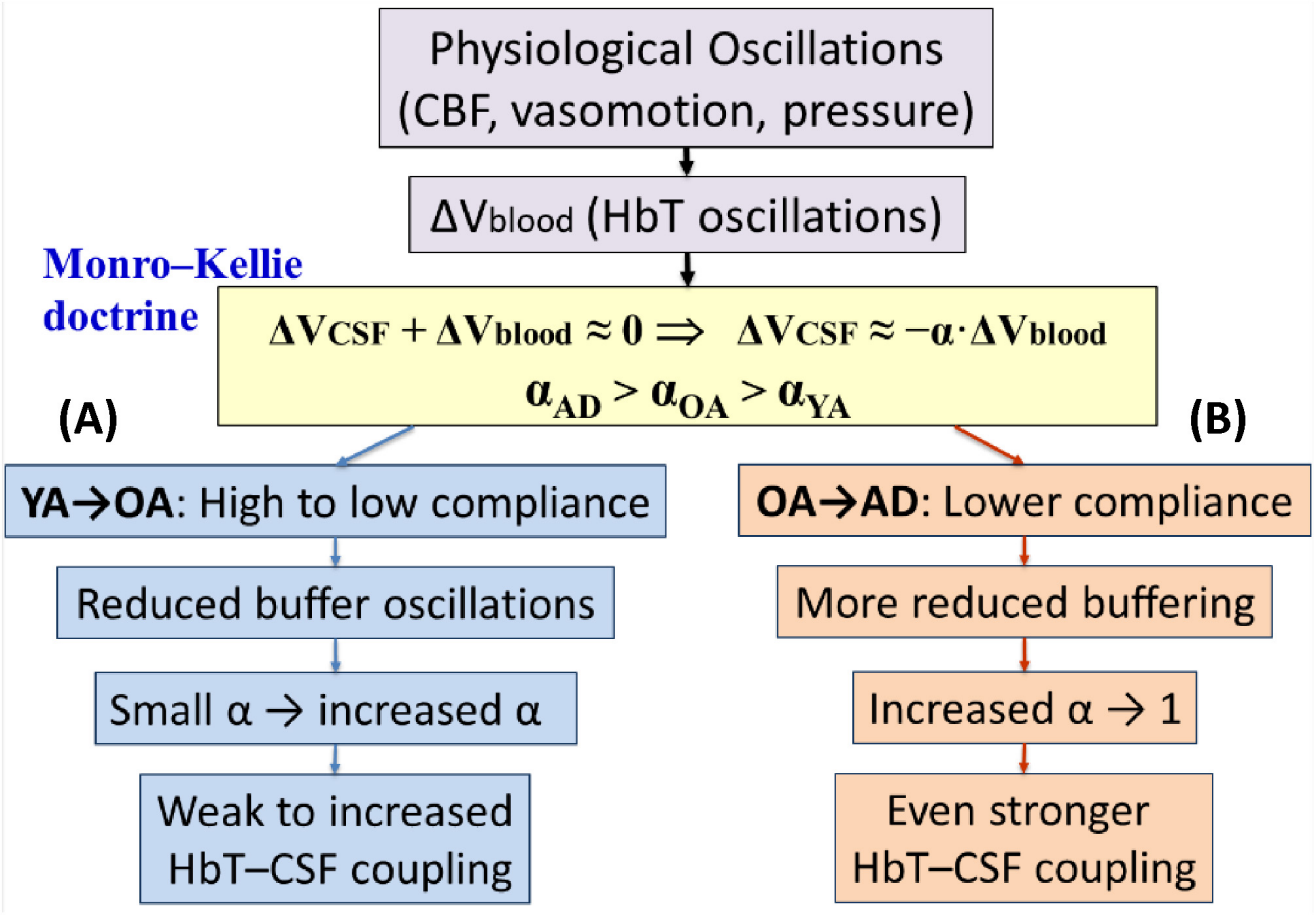
Mechanistic flow of increased inverse HbT-CSF coupling from YA to OA and to patients with early AD. Within the prefrontal cortex of the human brain, physiological oscillations arise from cerebral blood flow, slow and infraslow vasomotion, and blood pressure. These processes drive HbT oscillations that reflect changes in cerebral blood volume (Δ*V*_blood_). According to the Monro-Kellie doctrine, compensatory CSF volume changes (Δ*V*_CSF_) occur in response to a fraction (α, 0 = α = 1) of Δ*V*_blood_. **(A)** Reduced vascular compliance from YA to OA increases α, leading to stronger inverse HbT-CSF coupling. **(B)** Further increases in α in patients with AD, due to impaired compliance and intracranial compensation, result in even tighter coupling compared to OA. See text for details.

It follows Equation (12) shows that ΔHbT and ΔCSF oscillations become more tightly anti-correlated in OA than in YA. This same framework can be extended to interpret the increase trend of coupling from normal OA to AD. If AD is associated with further reductions in vascular compliance, impaired vasomotor buffering, or altered intracranial pressure-volume compensation, then αmay increase even further: namely, α_AD_>α_OA_>α_YA_.Figure 7 below schematically demonstrates the above-explained Mechanism.

Taking all the results from Figure 6 and mechanistic expectation from Figure 7 together, our findings suggest that cerebrovascular-CSF coupling strengthens progressively with aging and is further accentuated in early Alzheimer’s disease. The elevated coupling in OA likely reflects age-related stiffening of cerebral vessels, reduced intracranial compliance, and diminished efficiency of CSF regulation. The even stronger coupling observed in the AD group may indicate an early compensatory response to impaired vascular elasticity and glymphatic clearance, in which fluctuations in cerebral blood volume more directly drive CSF displacement.

### 4.4 Advances in the use of bbNIRS to investigate glymphatic dynamics

Previously reported methods used to demonstrate an inverse linear correlation between normalized Δ[HbT] and Δ[H_2_O]_*free*_ were based on regular NIRS using 4 NIR wavelengths (Myllylä et al., 2018; Borchardt et al., 2021), whereas we employed bbNIRS with 121 wavelengths (780–900 nm) in this study. In principle, 4 NIRS wavelengths are sufficient to quantify water content if 1-2 wavelengths lie near a characteristic water absorption band or peak (e.g., 930–950 nm or ∼980 nm). Using the modified Beer-Lambert law, absorption changes at these wavelengths can be used to solve for 3 chromophore concentrations (not including ΔCCOoxi), as demonstrated in prior studies (Myllylä et al., 2018; Borchardt et al., 2021). When measurements do not include the primary water absorption band (940–980 nm), accurate water quantification can still be achieved through multi-wavelength overdetermined fitting. Because our analysis used more than 100 wavelengths between 780 and 900 nm, the large spectral sampling provides sufficient constraints for the inverse solution, enabling reliable estimation of H_2_O content using the modified Beer–Lambert law. However, for non-broadband measurements using only 4 discrete wavelengths, inclusion of 1-2 wavelengths near the water absorption peak (∼980 nm) is recommended for accurate quantification of tissue H_2_O content.

### 4.5 Limitations and future work

While this paper revealed several novel findings on cerebrovascular–CSF coupling of the PFC in the human brain, we acknowledge several limitations and point out potential development for future investigations.

Technically, we did not utilize a short-separation probe for each bbNIRS channel, so we could not remove contaminants of signals from the superficial layers (i.e., the scalp and skull). All correlation metrics of cerebrovascular-CSF coupling in our study may have been underestimated because of the effects of extracranial tissues. Next, the sample size of patients with AD was relatively small, thus reducing the statistical power and generalizability of the results. In addition, we did not perform any cognitive assessments, such as the Mini-Mental State Examination (MMSE), to screen and evaluate the severity of AD in the patient group. While we subjectively estimated that all our AD participants were at an early stage, we did not have any objective measures to confirm this estimation. Therefore, the following improvements are suggested for future research: (1) it is technically advantageous and desirable to implement short- and long-separation probes in bbNIRS measurement settings that will enable the removal of signal contamination from extracranial layers; (2) more patients with AD should be recruited; and (3) all participants with or without AD should be assessed using quantitative cognitive measures, such as the MMSE. Moreover, it is highly desirable to perform MRI-based CSF or ventricular volume measurements that will strengthen mechanistic interpretations, as interpreted in this study.

## 5 Conclusion

Our findings in this study demonstrate that two-channel bbNIRS can noninvasively capture the cerebrovascular–CSF coupling dynamics of the human brain in aging and with Alzheimer’s disease. The progressively stronger inverse HbT-CSF coupling observed from YA to healthy OA and then to individuals with AD suggests that these anti-correlation-based metrics are sensitive to alterations in intracranial fluid regulation associated with aging and neurodegeneration. The strong cerebrovascular-CSF coupling observed in patients with AD may represent an early compensatory response to declining brain elasticity and imply impaired CSF drainage. However, the current analysis focused on infraslow oscillations (0.005–0.1 Hz), and our findings are best conceptualized as reflecting altered low-frequency cerebrovascular-CSF regulatory processes rather than direct measures of vascular compliance or glymphatic transport. These results may provide a potential complementary marker for the early detection of dementia-related disorders.

## Data Availability

The raw data supporting the conclusions of this article will be made available by the author, without undue reservation.
