# Supplementary material for: Cerebrovascular–CSF coupling measured by broadband near-infrared spectroscopy as a physiological marker of brain aging and Alzheimer’s disease
Source: Front Aging Neurosci. 2026 May 1;18:1757765. doi: 10.3389/fnagi.2026.1757765 (PMC13176143; doi:10.3389/fnagi.2026.1757765)
Supplement: Supplementary file 1 [file Supplementary_file_1.docx]

Supplementary Material

Cerebrovascular–CSF Coupling Measured by broadband near-infrared spectroscopy as a Physiological Marker of Brain Aging and Alzheimer’s Disease

**Authors:** Fiza Saeed *et al.*

*Corresponding author. Email: [hanli@uta.edu](mailto:hanli@uta.edu)

1. **Inclusion and exclusion criteria for both healthy older adults and patients with Alzheimer’s disease**

Inclusion Criteria of Healthy Adults: (1) aged 55 and older; (2) not experiencing cognitive declines; and (3) those who can travel without assistance for an IRB-approved research site on UTA campus.

Inclusion Criteria of AD Patients: (1) aged 55 years and older; (2) showing mild symptoms of dementia (early stage of dementia) with a physician’s diagnose note; (3) whose caregivers can provide transportation to the research site and remain with them during the hour-long process when the study subjects do not live in a long-term care facility (e.g. nursing homes, assisted living facilities, and group homes); (4) who live in a long-term care facility and have permission from the respective facility for us to visit the facility for testing.

Exclusion Criteria: (1) Brain injuries/surgeries in a year before the study; (2) those who can’t provide consent on their own.

1. **A Mathematical derivation from the raw NIR spectra to the quantification of Δ[HbO], Δ[Hb], Δ[CCO_oxi_], and Δ[H_2_O]**


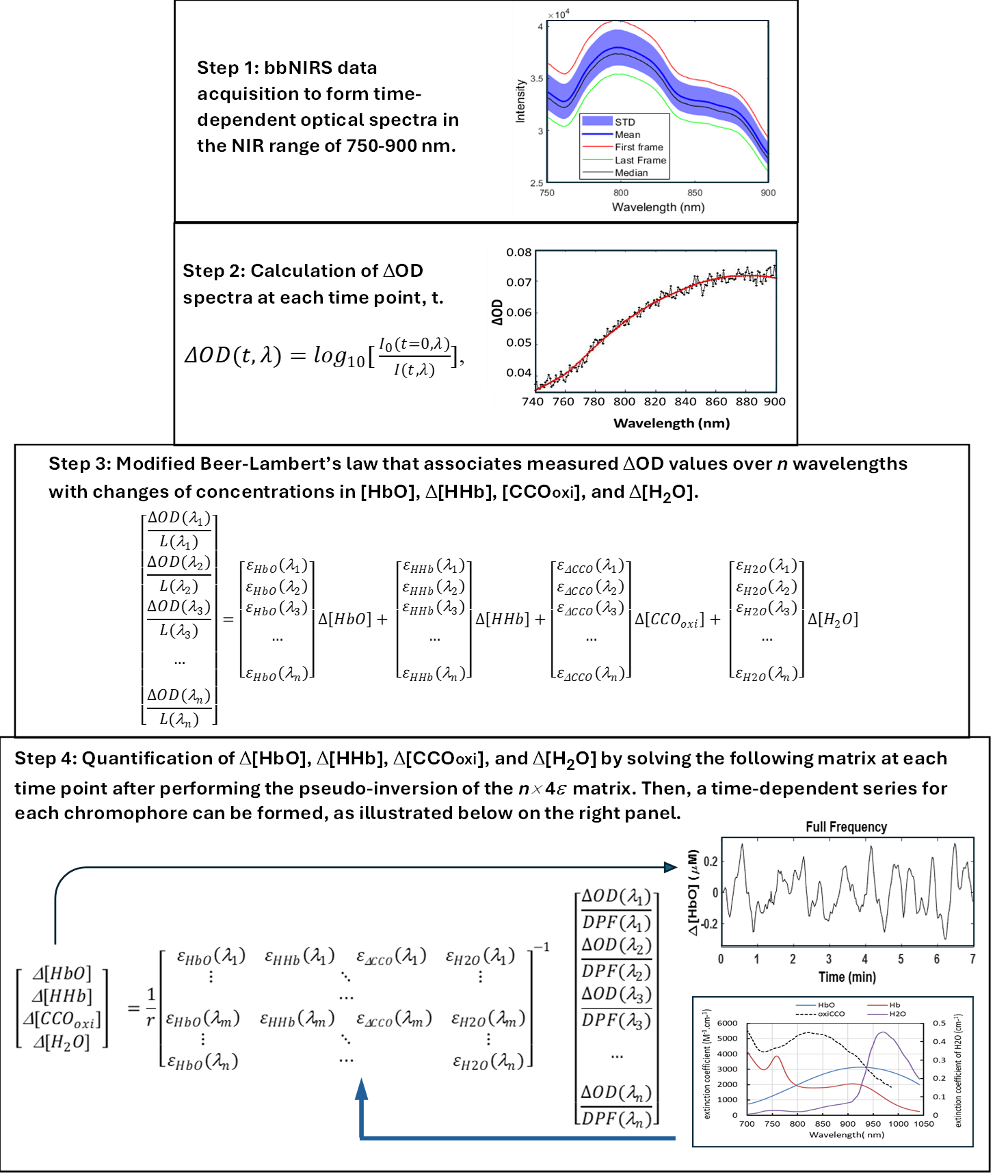


**Fig. S1** A data processing flowchart used to quantify Δ[HbO], Δ[HHb], Δ[CCOoxi], and Δ[H_2_O] from raw bbNIRS data. Δ[HbO] and Δ[HHb] are changes in oxy- and deoxy-hemoglobin concentrations; Δ[CCOoxi] represents changes in oxidized CCO concentration, and Δ[H_2_O] is changes in water percentage.

**Steps 1 and 2:**

A broadband near-infrared spectroscopy (bbNIRS) system provides measurements of optical spectra at different times (t), as expressed *I(t, λ)*. A relative optical density spectrum, *ΔOD*(*t, λ*), can be defined and calculated at each wavelength λ as [1, 2]:

$\Delta OD(t,\lambda)={log}_{10} [\frac{I_{0}(t=0, \lambda)}{I(t,\lambda)}]$, (1)

where *I_0_(t=0, λ)* can be the baseline spectrum at time *t*=0 or an average of several initial baseline spectral readings (i.e., the first two spectra collected in each experiment), and *I(t, λ)* represent time-varying spectra acquired at each time point throughout the entire experiment.

**Step 3**:

The estimations of Δ[HbO], Δ[HHb], Δ[CCOoxi], and Δ[H_2_O] from raw spectral data taken with bbNIRS throughout the experiment were based on modified Beer-Lambert’s law [3], which offers a quantitative relationship of ΔOD(λ) on Δ[HbO], Δ[HHb], Δ[CCOoxi], and Δ[H_2_O] at each wavelength, λ, at each time point, with a wavelength-dependent path-length factor, L(λ). Based on optical diffusion theory [4], ΔOD(λ)/L(λ) can be expressed as a sum of optical absorbance contributed by Δ[HbO], Δ[HHb], Δ[CCO], and Δ[H_2_O] components, as given below:

(2)

where Δ[HbO], Δ[HHb], Δ[CCO], and Δ[H_2_O] are relative concentration changes of HbO, HHb, oxidized CCO, and H_2_O, respectively; ε_HbO_(λ) and ε_HHb_(λ)) represent the extinction coefficients at each wavelength of HbO and HHb. ε_ΔCCO_(λ) is the difference extinction coefficient spectrum of cytochrome-c-oxidase. All of these ε values can be found in ref. [1]; L(λ) is a wavelength dependent factor that denotes the effective pathlength of the detected photons through tissues at each wavelength. According to the Modified Beer-Lambert Law [3, 5], L(λ) can be expressed as:

,

(3)

where *r* is a constant that denotes the source-detector distance. In this study, we used source detector separation of 3 cm, so *r*=3. The wavelength dependence of L(λ) is caused by a wavelength-dependent differential pathlength factor, DPF(λ).

**Step 4**:


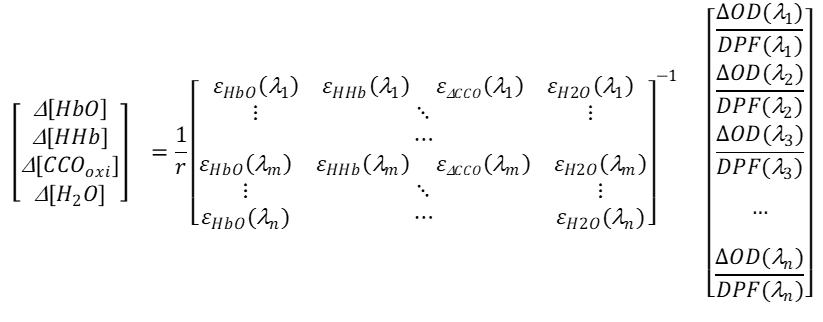
By substituting Eq. (3) into Eq. (2) for multiple wavelengths, the estimation of Δ[HbO], Δ[HHb], Δ[CCOoxi], and Δ[H_2_O] can be expressed as follows.

(4)

In order to accurately solve Δ[HbO], Δ[HHb], Δ[CCOoxi] and Δ[H_2_O] using Eq. (4), we would need to know DPF(λ) in the wavelength range of our measurements. It is known that appropriate or accurate selection/estimation of wavelength-dependent DPF is crucial for accurate estimation of chromophore concentrations [6]. In this study, DPF(λ) values were assumed to be time-invariant because of given stable brain optical properties. Based on diffusion theory with the semi-infinite boundary geometry [7], DPF(λ) can be determined by

(5)

$$DPF(\lambda)=\frac{\sqrt{3\mu_{s}'(\lambda)}}{2\sqrt{\mu_{a}(\lambda)}}*\frac{r\sqrt{3\mu_{a}(\lambda)\mu_{s}'(\lambda)}}{r\sqrt{3\mu_{a}(\lambda)\mu_{s}'(\lambda)}+1}$$

where µ_a_(λ) and µ_s_'(λ) are the estimated absorption and reduced scattering coefficients across the wavelength range of interest.

Values of µ_a_(λ) and µ_s_'(λ) were measured using a tissue oximeter (OxiplexTS, ISS) that operates in the frequency-domain. This device provides readings of *µ_a_* and *µ_s_'* values at 750 nm and 830 nm, as well as absolute concentrations of [HbO] and [HHb] [7]. However, to obtain µ_s_'(λ) values across the entire range of wavelengths from 780-900 nm, we used Mie theory to interpolate and extrapolate the two measured µ_s_' values at 750 nm and 830 nm. Mie theory is typically represented by kλ^-b^, where k and b were determined by fitting this equation to both *µ_s_'* values at 750 nm and 830 nm [8]. In addition, absorption coefficients in the same wavelength range (780-900 nm) were estimated based on [HbO] and [HHb] measured by the same tissue oximeter [4].

After combining the measured ΔOD(λ) values across the measurement period and empirical µa(λ) and µs'(λ) values of the human forehead [2], we were able to solve eq. (4) at each measurement time point using MATLAB, achieving temporal series of Δ[HbO], Δ[HHb] and Δ[CCOoxi] under respective experimental conditions. Specifically, our calculations covered the spectral range of 780-900 nm with a total of 121 wavelengths.

1. **Visualization of decomposition of a Δ[HbO] time series into three frequency bands**

**0.005-0.2 Hz original signal**

**Filtered E, N, and M signals**

**Fig. S2** Decomposition of a Δ[HbO] time series into three frequency bands

In S2, panel (a) shows an example of a 7-min Δ[HbO] time series from one of the two-channel bbNIRS datasets of a participant. Three curves in panel (b) were obtained after applying a Butterworth band-pass filter to the trace in (a) using separate bandpass filters in endogenic (E: 0.005-0.02 Hz), neurogenic (N: 0.02-0.04 Hz), and myogenic (M: 0.04-0.2 Hz) frequency ranges. This set of figures illustrates how different infraslow oscillation components contribute to the composition of the wideband (0.005–0.2 Hz) original signal.

**
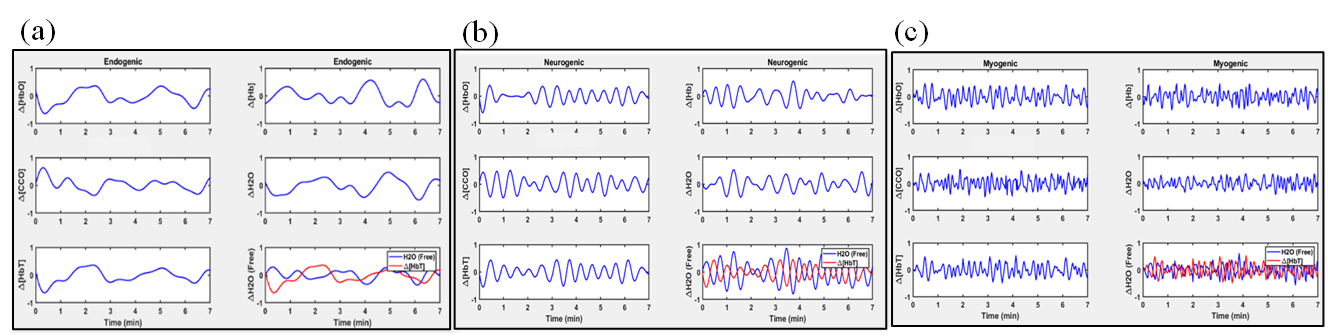
**

**Fig. S3** Time series of six neurophysiological quantities in three frequency bands

In each panel of Fig. S3, six sub-panels are 7-min time series of concentrations of oxy-hemoglobin (Δ[HbO]), deoxy-hemoglobin(Δ[Hb]), total hemoglobin (Δ[HbT] = Δ[HbO] + Δ[Hb]), oxidized cytochrome c oxidase (Δ[CCO_oxi_]), water content (Δ[H_2_O]), and free water content in CSF (Δ[H_2_O_free_]) in PFC of a participant. Panel (a) to (c) show the respective metrics in endogenic (0.005-0.02 Hz), neurogenic (0.02-0.04 Hz), and myogenic (0.04-0.1 Hz) frequency bands, respectively. The sub-panel on the right bottom shows temporal profiles of Δ[HbT] (red) and Δ[H_2_O_free_] (blue), exhibiting inverse oscillation pattens between them.

**Table S1** Statistical results of bilaterally averaged Fisher Z-transformed correlation coefficients between healthy young adults (YA; n = 26), healthy older adults (OA; n = 27), and AD patients (n = 16), obtained using one-way ANOVA followed by planned pairwise contrasts.

| **frequency** | **Endogenic** (0.005-0.02 Hz) | **Neurogenic** (0.02-0.04 Hz) | **Myogenic**  (0.04-0.1 Hz) |
| --- | --- | --- | --- |
| **YA vs OA** | 0.029 | 0.448 | 0.017 |
| **OA vs AD** | 0.028 | < 0.001 | 0.026 |
| **YA vs AD** | < 0.0001 | < 0.0001 | < 0.0001 |
|  |  |  |  |

**Table S2** Statistical results of bilaterally averaged slope coefficients between healthy young adults (YA; n = 26), healthy older adults (OA; n = 27), and AD patients (n = 16), obtained using one-way ANOVA followed by planned pairwise contrasts.

| **frequency** | **Endogenic** (0.005-0.02 Hz) | **Neurogenic** (0.02-0.04 Hz) | **Myogenic**  (0.04-0.1 Hz) |
| --- | --- | --- | --- |
| **YA vs OA** | 0.017 | 0.079 | 0.001 |
| **OA vs AD** | 0.018 | 0.021 | 0.125 |
| **YA vs AD** | < 0.0001 | < 0.001 | < 0.0001 |

**References:**

[1] C. Kolyva *et al.*, "Systematic investigation of changes in oxidized cerebral cytochrome c oxidase concentration during frontal lobe activation in healthy adults," *Biomed Opt Express,* vol. 3, no. 10, pp. 2550-66, Oct 1 2012, doi: 10.1364/BOE.3.002550.

[2] X. Wang *et al.*, "Up-regulation of cerebral cytochrome-c-oxidase and hemodynamics by transcranial infrared laser stimulation: A broadband near-infrared spectroscopy study," *J Cereb Blood Flow Metab,* vol. 37, no. 12, pp. 3789-3802, Dec 2017, doi: 10.1177/0271678X17691783.

[3] L. Kocsis, P. Herman, and A. Eke, "The modified Beer-Lambert law revisited," *Phys Med Biol,* vol. 51, no. 5, pp. N91-8, Mar 7 2006, doi: 10.1088/0031-9155/51/5/N02.

[4] X. Wang, F. Tian, S. S. Soni, F. Gonzalez-Lima, and H. Liu, "Interplay between up-regulation of cytochrome-c-oxidase and hemoglobin oxygenation induced by near-infrared laser," *Sci Rep,* vol. 6, p. 30540, 2016, doi: 10.1038/srep30540.

[5] F. Scholkmann *et al.*, "A review on continuous wave functional near-infrared spectroscopy and imaging instrumentation and methodology," *NeuroImage,* vol. 85 Pt 1, pp. 6-27, Jan 15 2014, doi: 10.1016/j.neuroimage.2013.05.004.

[6] S. J. Matcher, M. Cope, and D. T. Delpy, "Use of the water absorption spectrum to quantify tissue chromophore concentration changes in near-infrared spectroscopy," *Phys Med Biol,* vol. 39, no. 1, pp. 177-96, Jan 1994. [Online]. Available: <https://www.ncbi.nlm.nih.gov/pubmed/7651995>.

[7] S. Fantini *et al.*, "Non-invasive optical monitoring of the newborn piglet brain using continuous-wave and frequency-domain spectroscopy," *Phys Med Biol,* vol. 44, no. 6, pp. 1543-63, Jun 1999. [Online]. Available: <http://www.ncbi.nlm.nih.gov/pubmed/10498522>.

[8] S. L. Jacques, "Optical properties of biological tissues: a review," *Phys Med Biol,* vol. 58, no. 11, pp. R37-61, Jun 7 2013, doi: 10.1088/0031-9155/58/11/R37.
